# HIV-1 Tat promotes premature brain aging

**DOI:** 10.1186/1471-2334-14-S2-P63

**Published:** 2014-05-23

**Authors:** Asen Bagashev, Ruma Mukerjee, Jenny Shrestha, Maryline Santerre, Ying Wang, Bassel E Sawaya

**Affiliations:** 1 Temple University, Philadelphia, USA

## Background

It is estimated that by 2015, about half of all HIV-positive individuals will be older than 50 due to the introduction of the HAART. Yet those over 50 progress to AIDS faster than adults in their 20s or 30s, and those in the younger age bracket still exhibit illnesses and clinical conditions commonly associated with older people, such as HIV-associated neurocognitive disorders (HAND), certain cancers, liver and bones diseases. For the most part, the reasons for this have remained a mystery. In support of the eradication failure, studies showed the persistence of HIV-1 in brain cells as well as the presence of viral proteins in CSF. This notion was supported by the compelling neuropathological data suggesting that the loss of Synaptic Plasticity occurs with the ongoing presence of virus and despite HAART. Clinically, these neuropathological data manifest by a gradual loss of working memory and learning disability that may manifest by symptoms similar to the ones observed in aged brain. Anatomically, working memory and learning ability functions are assured by neurons of the hippocampus, a brain area known-to-be affected by HIV-1 proteins such as gp120.

## Results

Mechanistically, several laboratories, demonstrated that Tat protein performs its functions through deregulation of several molecular pathways that can deregulate the mitochondrial bioenergy such as depletion of mitochondrial calcium and release of ROS and decrease of ATP. Once altered, these factors cause disruption of mitochondria distribution along the axons leading to slower or loss axonal transport, thus, preventing neuronal communication. Interestingly, CREB protein has been shown to play a key role in these events directly. Similar data were obtained with tissues isolated from AD patients.

## Hypothesis

The most critical signaling pathway for the regulation of long-lasting memory formation is that containing CREB and its downstream targets. CREB is often referred to as “the master of memory genes.” Therefore, we will examine the impact of HIV-1 Tat protein on mitochondrial functions by focusing on the role of CREB protein.

## Expected outcome

Identification of the molecular mechanisms used by Tat leading to the development of Premature Brain Aging.

